# Supplementary figures and images for: Antioxidant Properties of Hydrogen Gas Attenuates Oxidative Stress in Airway Epithelial Cells
Source: Molecules. 2021 Oct 21;26(21):6375. doi: 10.3390/molecules26216375 (PMC8588133; doi:10.3390/molecules26216375)

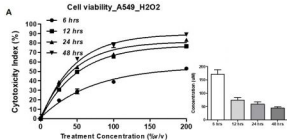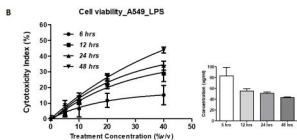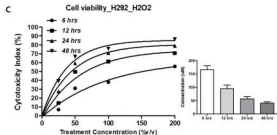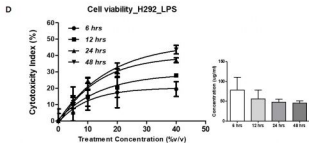

Supplement: Supplementary file 1 [file molecules-26-06375-s001.zip › molecules-1413885-Figure S1.pdf]
